# Validation of the rapid assessment procedure for loiasis (RAPLOA) in the democratic republic of Congo

**DOI:** 10.1186/1756-3305-5-25

**Published:** 2012-02-02

**Authors:** Samuel Wanji, Dowo O Akotshi, Maurice N Mutro, Floribert Tepage, Tony O Ukety, Peter J Diggle, Jan H Remme

**Affiliations:** 1 University of Buea, Faculty of Science, Department Microbiology and Parasitology, P.O. Box 63, Buea, Cameroon; 2 Research Foundation for Tropical Diseases and the Environment (REFOTDE), P.O. Box 474, Buea, Cameroon; 3 Programme National de Lutte contre l'Onchocercose, Ministère de la Santé Publique, Kinshasa, République démocratique du Congo; 4 Centre de Recherche en Maladies Tropicales de l'Ituri, Hôpital Général de Référence de Rethy, République démocratique du Congo; 5 Programme National de Lutte contre l'Onchocercose, Ministère de la Santé Publique, Buta, République démocratique du Congo; 6 World Health Organization, Prevention of Blindness and Deafness, Avenue Appia 20, 1211 Geneva 27, Switzerland; 7 Faculty of Health and Medicine, Lancaster University, Lancaster LA1 4YB, UK; 8 120 Rue des Campanules, 01210 Ornex, France

**Keywords:** RAPLOA, loiasis, ivermectin, onchocerciasis, lymphatic filariasis

## Abstract

**Background:**

A simple method called RAPLOA, to rapidly assess what proportion of people in a community are infected with *L. loa *and hence which communities are at high risk of severe adverse reactions following ivermectin treatment, was developed in Cameroon and Nigeria. The method needed further validation in other geographical and cultural contexts before its application in all endemic countries. The present study was designed to validate RAPLOA in two regions in the North East and South West of the Democratic Republic of Congo.

**Methods:**

In each study region, villages were selected from different bio-ecological zones in order to cover a wide range of loiasis endemicity. In each selected community, 80 people above the age of 15 years were interviewed for a history of eye worm (migration of adult *L. loa *under the conjunctiva of the eye) and parasitologically examined for the presence and intensity of *L. loa *infection. In total, 8100 individuals from 99 villages were enrolled into the study.

**Results:**

The results confirmed the findings of the original RAPLOA study: i) the eye worm phenomenon was well-known in all endemic areas, ii) there was a clear relationship between the prevalence of eye worm history and the prevalence and intensity of *L. loa *microfilaraemia, and iii) using a threshold of 40%, the prevalence of eye worm history was a sensitive and specific indicator of high-risk communities.

**Conclusion:**

Following this successful validation, RAPLOA was recommended for the assessment of loiasis endemicity in areas targeted for ivermectin treatment by lymphatic filariasis and onchocerciasis control programmes.

## Background

The control of onchocerciasis in Africa is based on mass treatment with ivermectin. Community-directed distribution of annual doses of ivermectin, introduced by the African Programme for Onchocerciasis Control (APOC), is an important component of the control strategy. Each community itself is in charge of designing and implementing the ivermectin treatment [1]. This strategy has been very successful and more than 65 million people in onchocerciasis endemic areas are treated annually with ivermectin [2,3].

However, several reports from Cameroon indicated that high microfilaraemia of *Loa loa *may be associated with severe and sometimes fatal encephalopathic reactions in patients who had taken ivermectin against onchocerciasis [4-6]. The risk of severe adverse reactions has been a major preoccupation for ivermectin treatment programmes throughout the central African sub-region where *L. loa *coexists with *Onchocerca volvulus *[7]. Several treatment programmes have been interrupted in Cameroon and the Democratic Republic of Congo (DRC).

The risk of severe adverse reactions to ivermectin treatment in *L. loa *infected individuals is related to the intensity of loiasis infection: the risk of developing marked or serious reactions is significantly higher when the *L. loa *exceeds 8,000 microfilariae/ml. The severity of adverse reaction becomes obvious in patients with more than 30,000 microfilariae/ml and the risk is very high for loads above 50,000 microfilariae/ml [5]. The prevalence of high microfilarial loads is closely related to the overall prevalence of microfilaraemia, and it has been proposed that a microfilarial prevalence of 20% be regarded as the threshold, above which there is an unacceptable risk of severe adverse reactions [8]. It is critical, therefore, that onchocerciasis control programmes identify such high-risk communities and withhold ivermectin treatment or make special provisions to ensure that severe adverse reactions are quickly detected and properly managed.

In the search for simple, non-invasive methods that can facilitate the large scale identification of high risk communities, a study carried out in Cameroon and Nigeria in 2001, supported by the UNDP/World bank/WHO Special Programme for Research and Training in Tropical Diseases (TDR) and APOC, led to the development of a Rapid Assessment Procedure for loiasis (RAPLOA) [9,10]. This method is based on a striking clinical manifestation of loiasis, the migration of the adult *L loa *worm under the conjunctiva of the eye. The major findings from the RAPLOA development study were the following: (i) the eye worm phenomenon is well known in *L. loa *endemic areas and people always have local names for it; (ii) there was a clear relationship between the percentage of community members that reported a history of eye worm and the community prevalence of loiasis infection; (iii) using a threshold of 40%, the prevalence of eye worm history was a good indicator of high-risk communities, i.e. communities where the prevalence of *L. loa *microfilaraemia was greater than 20%, the prevalence of high microfilarial loads (> 8,000 mf/ml) was greater than 5%, or the prevalence of very high microfilarial loads (> 30,000 mf/ml) was greater than 2%; (iv) the Rapid Assessment procedure of loaisis (RAPLOA) based on the prevalence of the history of eye worm had a high sensitivity and specificity for detecting high risk communities.

Based on these results, the independent expert committee of the African Programme for Onchocerciasis Control (APOC) endorsed the operational use of RAPLOA in Cameroon and Nigeria, but recommended further validation in other geographical areas to find out if those major findings are reproducible before the method could be recommended for all loiasis endemic countries. A validation study was therefore undertaken in two sites located at some 2000 km from each other in the South West and North East of the Democratic Republic of Congo.

Following the completion of the validation study in 2004, the results were reported to the expert committees of APOC and the Mectizan Donation Programme which jointly recommended the use of RAPLOA to estimate the prevalence of *L. loa *in all areas in Africa suspected to be endemic for this parasite [11]. APOC subsequently started a large-scale application of RAPLOA for mapping the distribution of loiasis endemicity in potentially endemic countries in Africa. Much of this mapping has now been completed and the results have begun to appear in the scientific literature [12,13], including a major publication with a comprehensive map of loiasis in Africa based on RAPLOA surveys in 4,798 communities in 11 potentially endemic African countries [14]. However, now that these mapping results are becoming widely available for operational decision-making for onchocerciasis control and lymphatic filariasis elimination programs in Africa, the question of the validity of the RAPLOA method is increasingly raised. It therefore became important to publish the results of the validation study in the scientific literature and provide the scientific community with the complete evidence on the validity of the RAPLOA method for estimating the prevalence of loiasis in different African countries. This article therefore provides a comprehensive report of the methodology and results of the validation study in two sites in the Democratic Republic of Congo.

## Methods

### Study design

The study was designed to study the relationship between the prevalence of eye worm history and parasitological indicators of loiasis infection, and to compare the results with the main findings of the earlier RAPLOA development study. The same methodology as in the previous study was applied, in which communities were selected from a wide range of loiasis endemicity levels. In each selected village 80 randomly selected people were interviewed using the standard RAPLOA methodology and a blood sample was examined for *L. loa *microfilariae.

The surveys were done between January and May 2004 by research teams made up of parasitologists, social scientists and epidemiologists. Each team was trained on the study methodology, the field data collection involving both parasitologic and social science methods, the microscopic processing of the thick blood smears and data management.

### Study sites

**Figure 1:**
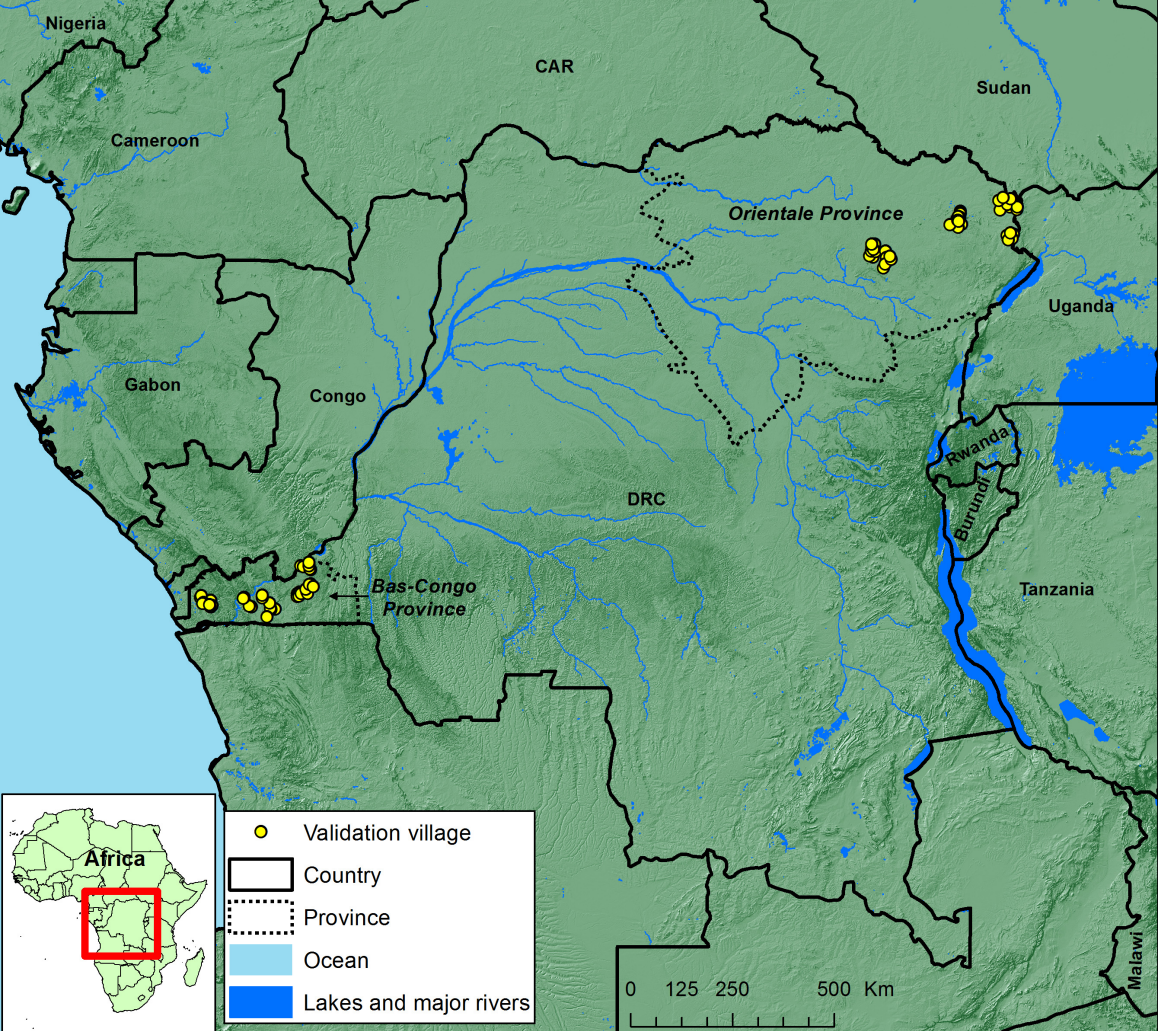
**Location of the validation villages in the Democratic Republic of Congo**.

The study was carried out in two sites of the Democratic Republic of Congo; the Bas-Congo and the Orientale provinces (Figure 1). In each site, study villages were selected from three different bio-ecological zones (forest, mosaic forest savannah and savannah), ranging from potentially high to potentially low endemicity levels for loiasis. The forest areas were Pawa and Wamba in the North East and Bas-fleuve (Mayombe) in the South West regions; 15 communities were to be surveyed in each of the forest areas. The savannah area involved Aru and Aba in the North East and Lukaya in the South West; 15 communities were to be surveyed in each of the savannah areas. The transition forest/savannah areas were made up of degraded forest with patches of gallery forests surrounded with vast savannah areas and involved Watsa in the North East and Cataracte in the South West; 20 communities were to be surveyed in each of the forest/savannah areas. Surveys were completed in all but one of the intended villages, giving data from a total of 99 villages for analysis.

### Study population

The study population consisted of males and females aged 15 years and above, who had been resident in the village for a minimum of five consecutive years and who had not taken antifilarial treatment within the year. Using a standard informed consent procedure, each selected individual was informed about the purpose of the study, the methods used, the voluntarily nature of participation and that they were free to refuse to participate. Every individual who consented to participate in the study signed an individual informed consent form and was given an identity code, interviewed for eye worm history and diagnosed parasitologically for *L. loa*. The investigation was carried out according to a research protocol approved by the Democratic Republic of Congo Ministry of Public Health and the Ethical Review Committee of the World Health Organisation.

### Rapid Assessment Procedure for Loiasis (RAPLOA)

The Rapid Assessment Procedure was based on the restricted definition of eye worm; i.e. the past experience of eye worm, confirmed by a photograph of an adult worm of *L. loa *in the white part of the eye and with the duration of the most recent episode being between 1 and 7 days [9,10]

Questionnaires were administered in the French language or in local languages (Lingala, Kikongo and Kiyombe for the South West, Swahili, Kibudu and Bangala for the North East study site). The interview process took place according to the RAPLOA guidelines [15].

A community questionnaire was used to brief key informants, including village heads, school teachers, health workers, patent medicine dealers and traditional healers, on the objectives and expected outcomes of the study and elicit the local names for eye worm.

For the individual questionnaire, all consenting eligible individuals in the household were questioned on their past experience of eye worm. If the answer was positive, the individual was shown a photograph of the eye worm and guided to identify the worm on the picture, followed by another question on the duration of the most recent episode. The photograph was the same photograph used in the development of RAPLOA. Individuals who reported to have experienced eye worm, who could recognise the worm on the photograph and who reported the duration of the last episode to be between 1-7 days were classified as RAPLOA positive and the rest were RAPLOA negative.

### Parasitological examination

Thick blood films to search for *L. Loa *microfilariae were prepared from a standardised 50 μl finger-prick blood sample, collected under aseptic conditions between 10:00 and 16:00 hours using a 75 μl non-heparinised capillary tube. The smear was prepared by spreading the blood on a clean slide over an area of 1.5 cm × 2.5 cm. Blood smears were de-haemoglobinised with tap water for 5 to 10 minutes, fixed with methanol for 1 minute and stained in 10% Giemsa for 45 minutes. The slides were read under a microscope at × 10. The microfilariae of *L. loa *were identified [16] and counted individually. The counts were expressed as microfilariae per milliliter (mf/ml) of blood.

### Data analysis

Data from the two study sites and from the earlier development study were analyzed using R Version 2.10.1 [17]. For each community the Rapid Assessment Procedures index was calculated as the proportion of people who reported past experience of eye worm, confirmed by means of a photograph of adult *L. loa *in the eye and that it lasted 1-7 days. Three parasitological indices were also calculated: prevalence of microfilaraemia in thick blood film; prevalence of high intensity of infection (> 8000 microfilariae/ml); prevalence of very high intensity of infection (> 30000 microfilariae/ml). Each index was analysed on the logit-scale to linearise the relationships amongst the four indices.

Linear regressions, weighted by the sample size in each community, were fitted to the relationship between the logit-transformed RAPLOA index and each of the parasitological indices. In each case, the single regression line for the complete validation data-set was compared with the two regression lines for the separate sites using a likelihood ratio test (F-test). The same method was then used to compare regression lines fitted to the complete validation data-set from Democratic Republic of Congo and the RAPLOA development data-set from Cameroon and Nigeria.

To assess the performance of a RAPLOA-based index against Mf prevalence thresholds to detect high risk communities, we defined the calibration points between the RAPLOA index and each of the parasitological indices by inspection of scatterplots. We then calculated the sensitivity and specificity of the RAPLOA index for detecting high risk communities, firstly by an in-sample method using the data from the two validation sites in DRC, then by an out-of-sample method using the data from the development study in Cameroon. The out-of-sample performance was also investigated using ROC curves over a wide range of calibration points, and the empirical optimum calculated as the value that minimized the distance between the ROC curve and the point (0,1), that represents perfect sensitivity and specificity.

## Results

Table 1 summarises the number of communities surveyed and numbers of people examined during the study. In all, 8100 individuals were examined from 99 communities, of which 49 were in South West DRC and 50 in North East DRC.

**Table 1:** Study population with prevalence RAPLOA, microfilaraemia, high microfilaraemia (> 8000 Mf/ml), very high microfilaraemia (30,000 Mf/ml) in different bio-ecological zones of the two validation study sites.

Study sites	Bio-ecological zones	Number of communities	Number of individuals examined	RAPLOA prevalence (%)	Prevalence microfilaraemia (%)	Prevalence microfilaraemia > 8000 mf/ml (%)	Prevalence microfilaraemia > 30,000 mf/ml (%)
South West DRC	Forest	14	1130	59.6	24.6	7.6	2.3
	
	Transition forest/savannah	20	1686	16.0	2.6	0.3	0
	
	Savannah	15	1193	27.5	14	4.1	0.6
	
	**Subtotal**	**49**	**4009**	**31.7**	**12.2**	**3.5**	**0.8**

North East DRC	Forest	15	1225	63.9	27.3	7.8	2.3
	
	Transition forest/savannah	20	1249	2.1	0.4	0	0
	
	Savannah	15	1617	17.9	4.0	1.1	0.3
	
	**Subtotal**	**50**	**4091**	**26.9**	**9.9**	**2.7**	**0.8**

Total		**99**	**8100**	**29.3**	**11.0**	**3.1**	**0.8**

### Knowledge of eye worm, attitude and practice in study communities

Local terms for eye worms were reported from both study sites; most were descriptive. Within the Kikongo language group of South West DRC, the local term was "nyaka sa meso" = worm of the eye. In Swahili and Kibula in North East DRC it was "upiyo" = caterpillar of the eye. In the forest areas in all the study sites, some communities had "eye worm specialists", who are capable of removing adult *L. loa *as they migrate across the sub-conjunctiva. More often, villagers used plant extracts to accelerate the migration of the worm across the eye.

### Level of endemicity of *L. loa *in different bio-ecological zones

The prevalence of *L. loa *infection varied considerably between bio-ecological zones within each study site. All of the forest zones were highly endemic for loiasis. In South West DRC, the prevalence of microfilaraemia in the forest villages had a range of 17.0-38.2, with a median of 23.3. In North East DRC, the microfilaraemia prevalence range was 8.8 to 38.5, with a median of 28.8. Transition forest savannah zones recorded a wide range of microfilaraemia prevalence between study sites. Savannah zones also recorded heterogeneity in prevalence, both in South West DRC [range 0-30.2, Median 13.6] and in North East DRC [range 0-2.5, Median 3.6]. The prevalences of high (> 8000 microfilariae/ml) and of very high (> 30,000 microfilariae/ml) intensity follow the pattern of the prevalence of *L. loa *microfilariae in different bio-ecological zones. In the forest areas, more than 7% of people examined harboured more than 8000 mf/ml and more than 2% of people examined harboured more than 30,000 microfilariae/ml in their blood. In the transition forest/savannah and in the savannah zones, the prevalences of high intensity and very high intensity of infection were in general lower.

RAPLOA prevalence was higher in the forest bio-ecological zones of both study sites: 59.6% in the South West DRC and 63.9% in the North East DRC. By comparison with the forest areas, RAPLOA prevalence was in general lower in the savannah and transition forest/savannah areas.

### Relationship between the prevalence and intensity of *L. loa *infection

**Figure 2:**
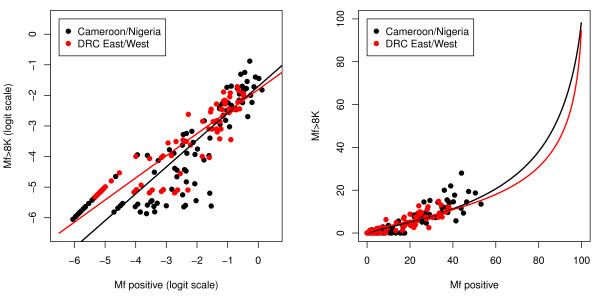
**Relationship between the prevalence of high microfilarial loads (> 8000 Mf/ml) and the prevalence of microfilaraemia at the community level (*original and validation data*)**. The black and red lines show the calibration models fitted to the original and validation data, respectively. The left-hand panel shows the data and models on the log-odds scale, the right-hand panel on the prevalence scale. The original data are from [10]).

Figure 2 shows the relationship between the prevalence and intensity of *L. loa *infection at the community level from the validation study along with the original data from Cameroon and Nigeria. The F-test for the difference between the fitted regression relationships in the two validation sites is not significant (p > 0.9). The F-test comparing the pooled data from the two validation sites and the original data from Cameroon and Nigeria is significant (p = 0.002), but the difference between the two is small, as shown by the two calibration curves in Figure 2. The prevalence of high intensity of *L. loa *infection (> 8000 microfilariae/ml) increased with the prevalence of *L. loa *microfilaraemia. The validation data confirmed previous findings that the 20% threshold prevalence of *L. loa *in thick smears at the community level corresponds to 5% and above of high intensity of infection.

### Relationship between RAPLOA prevalence and *L. loa *microfilaraemia

**Figure 3:**
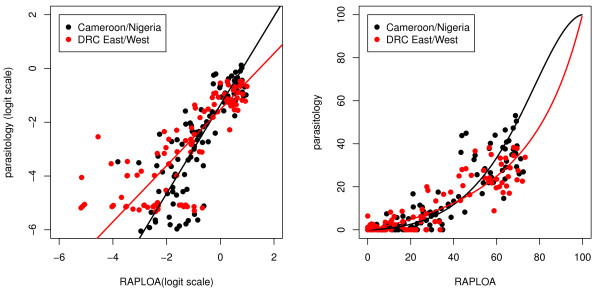
**Relationship between the prevalence of microfilaraemia and the prevalence of the history of eye worm (RAPLOA) (*original and validation data*)**. The black and red lines show the calibration models fitted to the original and validation data, respectively. The left-hand panel shows the data and models on the log-odds scale, the right-hand panel on the prevalence scale. The original data are from [10]).

**Figure 4:**
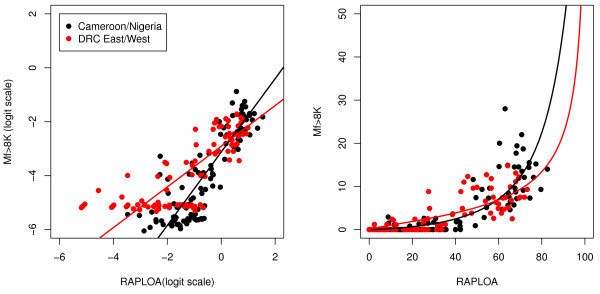
**Relationship between the prevalence of high intensity of microfilaraemia (> 8000 Mf/ml) and the prevalence of the history of eye worm (RAPLOA) (*original and validation data*)**. The solid line shows the regression model fitted to the validation data. The original data are from [10]).

**Figure 5:**
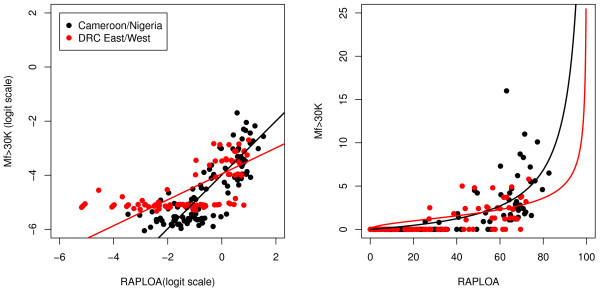
**Relationship between the prevalence of very high intensity of microfilaraemia (> 30,000 Mf/ml) and the prevalence of the history of eye worm (RAPLOA): *(original and validation data)***. The solid line shows the regression model fitted to the validation data. The original data are from [10]).

Figure 3 shows the relationship between the prevalence of eye worm (RAPLOA) and the prevalence of *L. loa *microfilaraemia. Once more, the F-test for the difference between the fitted regression relationships in the two validation sites is not significant (p = 0.594) whereas the difference between the pooled data from the two validation sites and the original data from Cameroon and Nigeria is significant (p < 0.001) but small in magnitude. A cut-off point of 40% prevalence of eye worm corresponds approximately to 20% prevalence of microfilaraemia in the validation study sites. We also used this same cut-off point to correspond to 5% prevalence of high intensity microfilaraemia (Figure 4) and to 2% prevalence of very high intensity microfilaraemia (Figure 5). In the case of very high intensity microfilaraemia, the linear regression model does not fit the data well but, as reported below, nevertheless still performs well as a prognostic tool.

### Sensitivity and specificity of RAPLOA with a threshold of 40%

**Figure 6:**
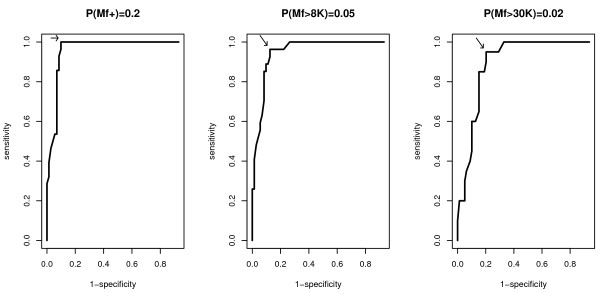
**ROC curves for the sensitivity and specificity of the RAPLOA index as a proxy for parasitological thresholds 20% positive Mf (left-hand panel), 5% Mf > 8 K (centre panel) and 2% Mf > 30 K (right-hand panel)**. The arrow on each ROC curve identifies the point closest to (0,1).

Table 2 shows the in-sample and out-of-sample sensitivities and specificities of the 40% cut-off value of the RAPLOA index as a proxy for all three of the parasitological thresholds: 20% positive Mf, 5% Mf > 8 K and 2% Mf > 30 K. Figure 6 shows the three corresponding out-of-sample ROC curves. The 40% RAPLOA cut-off is robust: all three out-of-sample sensitivities are greater than 90% and all three out-of-sample specificities are greater than 76%; the areas under the curves are 0.889, 0.880 and 0.832 and in all three cases, the point closest to (0,1) corresponds to a RAPLOA prevalence of 39%.

**Table 2:** Sensitivity and specificity of RAPLOA with threshold of 40% (*validation versus original data*)

Source of Data	Indicator of high risk of severe Adverse reactions	Sensitivity	Specificity
Validation study in DRC	Prevalence of *L. loa *Mf > 20%	100.0	90.1
	
	Prevalence of *L. loa *8,000+ Mf > 5%	96.3	87.5
	
	Prevalence of *L. loa *30,000+ Mf > 2%	95.0	79.7

Original study in Cameroon and	Prevalence of *L. loa *Mf > 20%	100.0	92.4
	
Nigeria [8]	Prevalence of *L. loa *8,000+ Mf > 5%	100.0	91.0
	
	Prevalence of *L. loa *30,000+ Mf > 2%	100.0	79.2

## Discussion

RAPLOA is a rapid assessment method of the prevalence of loiasis at the community level that was developed during a study in Cameroon and Nigeria [9,10]. Based on the results of this initial study, the independent expert committee of the African Programme for Onchocerciasis Control (APOC) endorsed the operational use of RAPLOA in Cameroon and Nigeria, but recommended further validation in other geographic areas and different socio-cultural contexts before the method could be applied in all endemic countries. The studies were therefore expanded to two study sites in the Democratic Republic of the Congo, which carries one-third of the global burden of onchocerciasis.

The two validation sites are located at a distance of some 2000 km from each other, in the Orientale Province in the North East of the Democratic Republic of Congo and in the Bas-Congo Province in the West of the country. The site in Orientale Province is near the eastern limits of the overall zone in Africa where loiasis may be endemic, while the site in Bas-Congo Province is in the South West of the loiasis zone [7,14]. Together with the original study sites in Cameroon and Nigeria, located in the North West of the loiasis zone, the RAPLOA sites provide a fair representation of the principal areas in Africa where loiasis is endemic.

Each of the two validation sites also covers a wide range of loiasis endemicity levels. The highest levels of endemicity were observed in the forest zones of the two study sites which could be explained by the abundant presence of *Chrysops *vectors which have numerous breeding sites in such forest regions [18-21]. The level of endemicity was much lower in the transition forest/savannah and savannah bio-ecological zones. Nevertheless, in several locations in the savannah and transition zones, relatively high endemicity of loiasis was recorded. This was seen in the South West DRC, not far from the city of Kinshasa, and in the North East DRC (Uelés region). The presence of loiasis in the savannah and transition zones is a result of hillside forests and gallery forests along the important rivers [22].

One of the outstanding questions after the development of RAPLOA was to know how far this method could remain useful in the prediction of the endemicity of loiasis in socio-cultural contexts different from those in which it was developed. The RAPLOA method is based on the history of clinical manifestations and largely depends on the appreciation of the eye worm phenomenon by the members of a community. The results of our validation study confirmed previous observations that the experience of eye worm migration is well known by members of endemic communities irrespective of their cultures and dialects [10,23,24] and that they have descriptive local names for the phenomenon. The subconjunctival migration of adult *L. loa *is a spectacular phenomenon in endemic communities, which are often located far from any health facility. This situation has even produced local "eye worm specialists" who are able to remove the migrating worms from the eye using a special tree spine, or a plant extract to accelerate the disappearance of worms from the sub-conjunctiva tissue.

The RAPLOA tool consists of just three very simple questions, including "have you ever had the condition shown in this picture?" asked while showing a clear photograph of a worm moving across the white part of an eye. Based on the response to these three questions by a sample of adults, the prevalence of the history of eye worm, or RAPLOA prevalence, is calculated for each community as an indicator of the level of loiasis endemicity. The study has confirmed that there exists a close relationship between the RAPLOA prevalence and parasitological indicators of loiasis endemicity at the community level, and that the RAPLOA prevalence can be used to identify communities where there is a high risk for adverse reactions to ivermectin treatment, i.e. communities where the prevalence of microfilaraemia is greater than 20%, the prevalence of high intensity (8,000 mf/ml) of *L. loa *infection is greater than 5%, or the prevalence of very high (30,000 mf/ml) infection is greater than 2%. The optimal diagnostic threshold for RAPLOA prevalence was 39% in the validation study, a result that is nearly identical to the 40% obtained in the original study. The sensitivity and specificity of a RAPLOA prevalence of 40% for the diagnosis of high-risk communities were high, with the sensitivity ranging from 95% to 100% and the specificity from 80% to 90%, and these figures are very similar to the sensitivity and specificity estimates obtained in the original study.

## Conclusion

The results of the validation study in the two sites in the Democratic Republic of Congo confirm the main findings of the original study in Cameroon and Nigeria, and the study has therefore validated the RAPLOA method for the rapid assessment of the prevalence of loiasis. The method is shown to produce consistent results in different geographic and social cultural settings, and to work as well as the much more cumbersome and invasive finger-prick thick blood film parasitological method.

## List of abbreviations

**APOC**: African Programme for Onchocerciasis Control; **DRC: **Democratic Republic of Congo; **RAPLOA**: Rapid Assessment Procedure for Loiasis; **TDR**: UNDP/World Bank/WHO Special Programme for Research and Training in Tropical Diseases; **Mf: **Microfilariae; **Mf positive**: Microfilarial positive; **Mf > 8 K**: Microfilarial load greater than 8000 microfilariae per milliliter of blood; **Mf > 30 K: **Microfilarial load greater than 30000 microfilariae per milliliter of blood.

## Competing interests

The authors declare that they have no competing interests.

## Authors' contributions

**SW **participated in the design of the study, the collection of data, the analysis of data and the drafting of the manuscript. **DOA **participated in the data collection and analysis. **MNM **participated in the data collection and analysis. **FT **participated in the data collection and analysis. **TOU **participated in the design of study and data analysis. **PJD **participated in the data analysis, and edited the manuscript. **JHR **participated in the study design, analyzed the data, interpreted the results and edited the manuscript. All authors read and approved the final version of the manuscript.
